# Extension of the COG and arCOG databases by amino acid and nucleotide sequences

**DOI:** 10.1186/1471-2105-9-479

**Published:** 2008-11-13

**Authors:** Florian Meereis, Michael Kaufmann

**Affiliations:** 1 The Protein Chemistry Group, Witten/Herdecke University, Stockumer Str. 10, 58448 Witten, Germany

## Abstract

**Background:**

The current versions of the COG and arCOG databases, both excellent frameworks for studies in comparative and functional genomics, do not contain the nucleotide sequences corresponding to their protein or protein domain entries.

**Results:**

Using sequence information obtained from GenBank flat files covering the completely sequenced genomes of the COG and arCOG databases, we constructed NUCOCOG (nucleotide sequences containing COG databases) as an extended version including all nucleotide sequences and in addition the amino acid sequences originally utilized to construct the current COG and arCOG databases. We make available three comprehensive single XML files containing the complete databases including all sequence information. In addition, we provide a web interface as a utility suitable to browse the NUCOCOG database for sequence retrieval. The database is accessible at .

**Conclusion:**

NUCOCOG offers the possibility to analyze any sequence related property in the context of the COG and arCOG framework simply by using script languages such as PERL applied to a large but single XML document.

## Background

**Figure 1:**
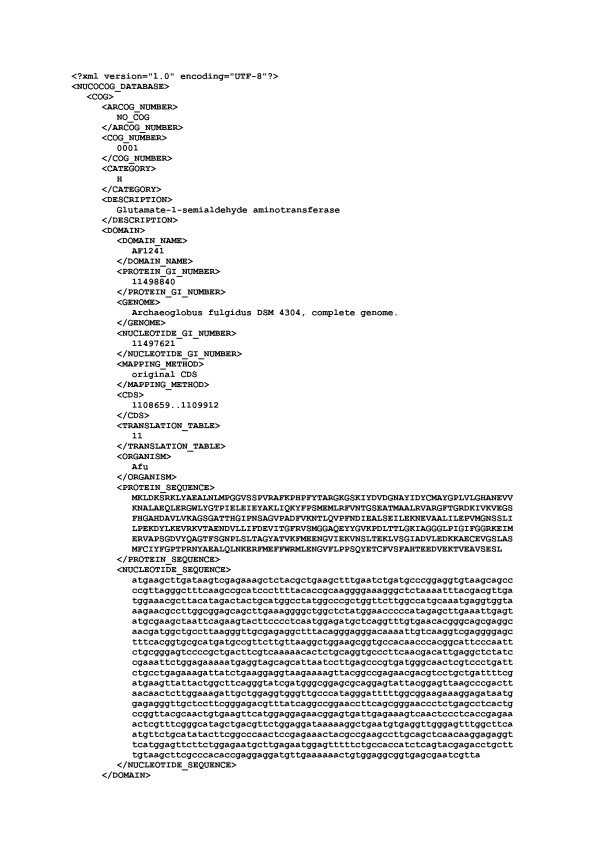
First lines of the NUCOCOG 242 MB XML file.

**Figure 2:**
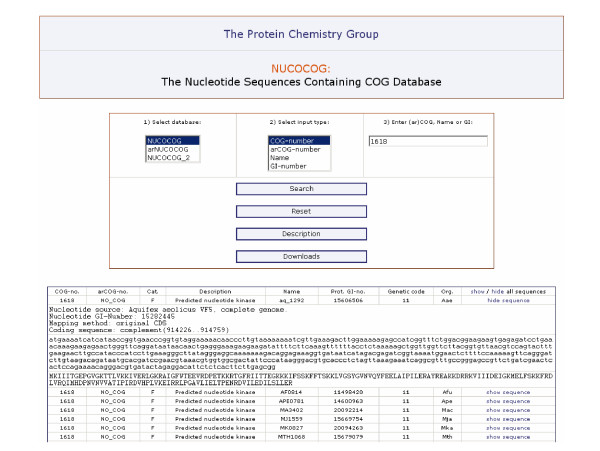
**Screenshot of the NUCOCOG retrieval utility at**[21].

The concept originally introduced by Tatusov *et al. *in 1997 [1] to assign protein sequences based on sequence similarities to COGs led to the establishment of the COG database which was updated repeatedly when more and more completely sequenced genomes became available [2-4]. During the last decade, the COG database became a distinguished tool in comparative and functional genomics [5]. The recently published Archaeal Clusters of Orthologous Genes (arCOG) are a refinement and update of archaeal sequences using a new sophisticated computational pipeline [6]. However, although the original protein sequences used to construct the databases are available via FTP [7,8], there are no direct assignments of protein or protein domain sequences to entries within the databases, and nucleotide sequences are completely absent. Since the COG and arCOG databases are excellent frameworks to study sequence specific aspects such as amino acid composition, GC content, codon usage or the like in both a functional and a phylogenetic context, versions including sequence information directly linked to every protein or protein domain were a desirable improvement. Here we present the latest update of the COG database, the arCOG database, and a combination of them as XML files (nucocog.xml, arnucocog.xml, and nucocog_2.xml, respectively) (Figs 1 and 2) that include both amino acid and nucleotide sequences directly assigned to their respective protein names and GI-numbers.

## Construction and content

### Construction of the NUCOCOG database

The NUCOCOG database (nucocog.xml) was constructed by repeatedly running a set of different PERL scripts to read, create and manipulate files containing ASCII data. All files containing the information required to create the NUCOCOG database were obtained via FTP from the NCBI. Three files related to the COG-database (*whog*, *myva*, *myva=gb*) were taken from [7] and 159 GBK files containing the genome information of the 66 organisms currently present in the COG database were taken from [9]. To obtain a maximum quality of nucleotide sequences we used current GBK files rather than the outdated ones corresponding to the original protein sequences. In principle, this may lead to errors due to the methods described below. However, those errors are very improbable and, if occurring at all, are negligible compared to all the ambiguous amino acids and nucleotides present even in current GBK versions (see table 1). The NUCOCOG database was built up by the following five main procedures: (i) The complete COG database as assembled in *whog*, was converted to an XML file which served as the scaffold that was subsequently stepwise extended to eventually represent the final NUCOCOG database. (ii) The amino acid sequences were extracted from *myva *by searching for their respective protein names as the unambiguous search keys. (iii) In the same manner, GI-numbers for all *complete *protein sequences were obtained from *myva=gb*. However, no protein *fragments *(domains) at all could be detected because their names are extended by an underscore followed by a consecutive number in *whog *which is not the case in *myva=gb*. We derived GI-numbers for those entries during a second run after the extensions of their names were truncated. (iv) Nucleotide sequences were then included separately for each of the 66 organisms by exclusively searching for organism specific sequences only in GBK files associated with the respective query organism. The amino acid sequences as annotated in *myva *were used as search keys to locate their corresponding nucleotide sequences in the GBK files. To detect all sequences, it was necessary to apply four different search approaches. The vast majority (98.4 %) of all nucleotide sequences were extracted by searching for matching amino acid sequences as annotated in the GBK files. Because some of the GBK files used in this work have been updated since the latest release of the COG database not every sequence could be detected by this method. We discovered additional sequences (1.4 %) by searching for matches with conceptual translated nucleotide sequences. Those sequences were located and their reading frames were determined according to the CDS information in the respective GBK file. In addition, the nucleotide sequences were extended by 300 adjacent nucleotides derived from the genome sequence in both directions prior to translation. Further sequences (0.1 %) could be found by searching for matching amino acid sequences in translations of whole genomes in all six reading frames. The residual 121 missing nucleotide sequences were included by manually editing the first qualifiers of the CDS feature key in the respective GBG file followed by a further search in conceptually translated nucleotide sequences. To inspect and edit GBK files, the genome annotation tool ARTEMIS [10,11] was used in combination with the pairwise sequence alignment software JAligner [12]. Mainly, we deleted annotated frameshifts, included new frameshifts and in some cases replaced mismatching amino acids by using coding information of the nearest matching amino acid sequence within the same reading frame. For each nucleotide sequence the respective mapping method was included to the XML-files *i. e.*"original CDS", "extension of original CDS", "conceptual translation of whole genome", or "created or edited CDS manually". (v) After this first version of the complete NUCOGOG database had been finished, some PERL scripts were run for verification and validation purposes with the main focus on translating the nucleotide sequences and comparing the translations to their respective annotations. For four entries (MK0324, MK0315, MK0689, MK0809) even the manual search for more information *e. g. *by BLAST [13] assigned no GI-number and we adopted the entry "gi?" from *myva=gb*.

**Table 1:** Content of the three NUCOCOG databases

	NUCOCOG	arNUCOCOG	NUCOCOG_2
domain sequences	144,320	81,616	204,890
Nucleotides	142,675,176	72,324,636	195,633,198
stop codons	94	62	115
a. a. a.: B	41	-	41
a. a. a.: U	24	27	42
a. a. a.: X	1,243	89	1,288
a. a. a.: Z	12	-	12
a. n.: b	9	-	9
a. n.: d	9	-	9
a. n.: h	4	-	4
a. n.: k	189	-	189
a. n.: m	163	3	164
a. n.: n	195	110	301
a. n.: r	328	1	328
a. n.: s	258	-	258
a. n.: v	7	-	7
a. n.: w	113	-	113
a. n.: y	660	3	660

The abbreviations used are a. a. a. for ambiguous amino acids and a. n. for ambiguous nucleotides.

### Construction of the arNUCOCOG database

The arNUCOCOG database (arnucocog.xml) was essentially constructed as described above using the information from ar40.fa and arCOG.csv [8] to build the initial XML-file. The current version of arCOGs includes the genome of *Thermoproteus tenax *which has not been published at the time of its release and by request of the sequencing consortium those proteins were removed from the ar40.fa file and are also not contained in the arNUCOCOG database. In addition, 34 sequences listed in arCOG.csv could not be located in ar40.fa. These proteins for various reasons were not translated and the authors detected them by tBLASTn, using an orthologous sequence from a close relative as a query (Kira Makarova, personal communication). We included those sequences manually by reproducing her work. Searching for matching amino acid sequences in the GBK files resulted in including 99.8 % of all nucleotide sequences. The remaining sequences were detected by the alternative methods described above and only three sequences needed to be searched manually. Many of the arCOGs are new and consequently not assigned to a classical COG-number. In all those cases, we included "NO_COG" between the respective tags. Because arCOG.csv contains protein gi-numbers as the domain-ids, no unique domain-ids are assigned to all split sequences. We improved this situation by adding consecutively numbered suffixes to those gi-numbers separated by an underscore *e.g. *<DOMAINNAME>118430839_1</DOMAINNAME>.

### Combining NUCOCOG with arNUCOCOG (NUCOCOG_2)

We also combined NUCOCOG and arNUCOCOG resulting in nucocog_2.xml. For that purpose, we removed all sequences from the 13 archaeal genomes from NUCOCOG and included all data from arNUCOCOG instead. In addition, we added those sequences from ar40.fa that according to the information from arCOG.csv are assigned to classical COGs but are not part of any arCOG. Finally, for those amino acid sequences their corresponding nucleotide sequences were included as described above and "NO_COG" was written between the arCOG-tags.

### Content of the NUCOCOG database

The content of the three database files is summarized in table 1. As can be seen, some nucleotide sequences contain stop codons within coding regions and there are both ambiguous amino acids (a. a. a.) and ambiguous nucleotides (a. n.). Consequently, in those cases a distinct translation of a codon to an amino acid is impossible. For that reason, although resulting in larger database files containing redundancies, we did not delete the amino acid sequences from our files after the databases had been constructed.

## Utility

Most of the users will probably use the set of databases according to the aim we primarily constructed it for:*i. e. *by downloading the XML files and analyzing them with respect to their own research questions and their individually developed software tools. Nevertheless, we also provide a web based utility to browse the databases for sequence retrieval by COG-number, arCOG-number, protein name, and GI-number. For that purpose, we used the Apache HTTP Server and an SQL backend. The XML files were converted to tables of an SQL database, one for all COG data and the other ones for the nucleotide and amino acid sequences, respectively. The names of the protein or protein domains were used as unique keys. Queries can be made by using the frontend written in PHP providing the option to select certain entries for displaying their corresponding amino acid and nucleotide sequences.

## Discussion

The COG and arCOG databases represent excellent collections of proteins (or protein domains). The version presented here including amino acid and nucleotide sequences allows answering all sequence related questions with respect to orthologous proteins *i. e. *proteins that are assumed to exhibit identical functions. For instance, one may ask whether enzymes involved in a certain metabolic pathway have constraints in their amino acid composition. This is described for enzymes involved in tryptophan biosynthesis since the 5 protein chains of the *E. coli trp *operon contain only 5 tryptophan residues [14]. Indeed, the "cognate bias hypothesis" stating that early in evolutionary history the biosynthetic enzymes for amino acid × gradually lost residues of × [15] could elegantly be tested using the NUCOCOG files presented here. Questions related to deviations of nucleotide sequence compositions such as codon usage or GC-content in dependence on the functions of the respective proteins could also be answered by exploring the XML files provided here. Furthermore, the COG framework had proved to be a powerful tool in conjunction with phylogenetic protein sequence distributions [16,17]. The possibility to examine clade specific features of nucleotide or amino acid sequences within the COG context could also uncover more precise data than those made available by simply comparing the sequences of whole genomes. For example, there are several studies dealing with differences in sequence specific properties between (hyper)thermophiles and mesophiles by comparing the sequence data of their complete genomes [18-20]. Those surveys do not account for possible differences in sequence signatures that depend solely on the function of the respective protein rather than the phylogenetic relationship of the organisms under investigation. To refine such studies, only proteins derived from different organisms but exhibiting identical biochemical functions should be compared on a large scale rather than just comparing complete genomes. With that intention we constructed NUCOCOG and our future work will exactly deal with the refinement described here of detecting thermophile-specific sequence signatures considering possible distortions due to comparing functionally different proteins.

## Conclusion

NUCOCOG is a version of the current COG and arCOG databases assembled in single XML files containing both amino acid and nucleotide sequences associated to their respective entries. In depth analysis of this XML files makes it possible to investigate any sequence specific property in the COG context, taking into account functional and phylogenetic relationships.

## Availability and requirements

The NUCOCOG database can be browsed by any web-browser at . [21]. In addition, the databases as three XML files and the source codes are freely available at the same URL.

## Abbreviations

ASCII: American standard code for information interchange; BLAST: basic local alignment search tool; CDS: coding sequence; COG: cluster of orthologous groups; FTP: file transfer protocol; GBK: GenBank (file-extension *.gbk*); GC: guanine-cytosine; GI: geninfo identifier; HTTP: hypertext transfer protocol; MB: megabyte; NCBI: National Center for Biotechnology Information; NUCOCOG: nucleotide sequences containing COG; PERL: practical extraction and report language; PHP: PHP hypertext pre-processor; SQL: structured query language; URL: uniform resource locator; XML: extensible markup language

## Authors' contributions

FM wrote the web-interface and implemented the databases on our server, MK constructed the NUCOCOG-database, conceived of the study and wrote the manuscript. Both authors read and approved the final manuscript.
